# Change in the Proportion of Death at Home during the COVID-19 Pandemic and Its Associated Factors in the Municipality Level: A Nationwide Study in Japan

**DOI:** 10.31662/jmaj.2023-0165

**Published:** 2024-03-18

**Authors:** Yu Sun, Masao Iwagami, Ryota Inokuchi, Nobuo Sakata, Tomoko Ito, Yuta Taniguchi, Satoru Yoshie, Nanako Tamiya

**Affiliations:** 1 Department of Health Services Research, Institute of Medicine, University of Tsukuba, Ibaraki, Japan; 2 Department of Primary Care and Medical Education, Institute of Medicine, University of Tsukuba, Ibaraki, Japan; 3 Health Services Research and Development Center, University of Tsukuba, Ibaraki, Japan; 4 Heisei Medical Welfare Group Research Institute, Tokyo, Japan; 5 Department of Home Healthcare, Setagaya Memorial Hospital, Tokyo, Japan; 6 Graduate School of Comprehensive Human Sciences, University of Tsukuba, Ibaraki, Japan; 7 Institute of Gerontology, University of Tokyo, Tokyo, Japan; 8 Institute for Future Initiatives, University of Tokyo, Tokyo, Japan; 9 Department of Health Policy and Management, School of Medicine, Keio University, Tokyo, Japan; 10 School of Medicine, Hiroshima University, Hiroshima, Japan

**Keywords:** home healthcare services, COVID-19, home deaths

## Abstract

**Introduction:**

The coronavirus disease 2019 (COVID-19) pandemic may have led to an increase in home deaths due to hospital bed shortage and hospital visitation restrictions. This study aimed to examine changes in the proportion of home deaths before and after the COVID-19 pandemic and identify associated factors.

**Methods:**

We used publicly available nationwide data to describe the proportion of home deaths among total deaths from 2015 to 2021. Furthermore, we used municipal-level data to examine the factors associated with the increase in the proportion of home deaths from 2019 to 2021. The dependent variable was the absolute change in the proportion of home deaths from 2019 to 2021. The independent variables included each municipality’s 2019 home death percentage, medical and long-term care (LTC) resources divided by the population of older people, population density, and cumulative number of COVID-19 cases. A multivariable linear regression analysis was conducted after the standardization of each variable.

**Results:**

The proportions of home deaths in 2015, 2019, and 2021 were 12.7%, 13.6%, and 17.2%, respectively, indicating a sharp increase in home death rate after the COVID-19 pandemic. In the multivariable linear regression analysis that included 1,696 municipalities, conventional home care support clinics and hospitals (HCSCs) (coefficient [95% confidence intervals (CIs)], 0.19 [0.01-0.37]), enhanced HCSCs (0.53 [0.34-0.71]), home-visiting nurses (0.26 [0.06-0.46]), population density (0.44 [0.21-0.67]), and cumulative COVID-19 cases (0.49 [0.27-0.70]) were positively associated with the increase in home deaths, whereas beds of LTC welfare facilities (−0.55 [−0.74-−0.37]) and the proportion of home deaths in 2019 (−1.24 [−1.44-−1.05]) were negatively associated with the increase.

**Conclusions:**

During the COVID-19 pandemic, home deaths significantly increased, particularly in densely populated areas with high cumulative COVID-19 cases. HCSCs, especially enhanced HCSCs, are crucial for meeting the demand for home-based end-of-life care.

## Introduction

Coronavirus disease 2019 (COVID-19), a highly infectious and potentially fatal disease, has rapidly spread worldwide since a local outbreak in Wuhan, China, in December 2019 ^(1)^. Since the first COVID-19 case in Japan was recorded on January 16, 2020, the disease has continued to spread, with five pandemic waves by the end of 2021, which resulted in a cumulative total of 1.7 million cases and 18,000 deaths ^(2)^. The shortage of available hospital beds, owing to the categorization of COVID-19 as a Category II Infectious Disease in the Infectious Disease Control Law, along with the difficulties in bed management and staffing, led to a medical crisis ^(3)^. Especially during the fourth and fifth waves (from April to September 2021), patients with mild-to-moderate cases of COVID-19 could no longer be transferred to a specialized hospital after worsening, and patients at home or in designated accommodations could not be admitted to any hospital ^(3), (4)^. Furthermore, the COVID-19 pandemic prompted widespread visitation restrictions in almost all hospitals and facilities for the elderly to prevent infection from visitors. A survey conducted in October 2021 showed that 99% of palliative care units had enforced visitation restrictions ^(5)^.

Under these circumstances, several studies have reported that the COVID-19 pandemic has changed the end-of-life (EOL) care in Japan ^(6), (7)^ and in several countries worldwide ^(8), (9)^. A questionnaire survey administered to home-visit facilities showed that the number of patients dying at home and newly requesting home healthcare increased after the COVID-19 pandemic, particularly among those with cancer, respiratory disease, and dementia. Another study demonstrated that prefectures with a higher number of COVID-19 cases had a higher proportion of home deaths among patients with cancer, suggesting that COVID-19 increased the need for at-home EOL care among patients with cancer. The main reasons for the increased demand for home-based EOL care were considered to be the shortage of available hospital beds, particularly in the palliative care unit, and restrictions on inpatient visitation. These factors have prevented patients from spending valuable time with their loved ones during their final days ^(6), (7), (8)^.

However, these studies were based on questionnaires or focused exclusively on patients with cancer; thus, changes in the place of death in Japan as a whole need to be elucidated. Furthermore, while regional differences may exist due to variations in healthcare resources and population density, no study has investigated the geographic impact of the increase in home deaths during the COVID-19 pandemic, including healthcare resources and population density.

To address this knowledge gap, the present study aimed to describe changes in the proportion of home deaths during the COVID-19 pandemic and investigate the potential factors associated with this increase using nationwide data in Japan.

## Materials and Methods

### Home healthcare in Japan

All Japanese citizens have medical care coverage under a universal health insurance system, which includes occupational insurance for employees, National Health Insurance for the self-employed and retirees below 75 years old, and a late-stage medical care system for those aged 75 years and above ^(10), (11)^. In Japan, home healthcare involves regular home visits by physicians that are covered by health insurance. In 2006, the Ministry of Health, Labour and Welfare (MHLW) introduced home care support clinics and hospitals (HCSCs) ^(12)^. HCSCs offer a 24-h home-visiting care system upon the patient’s request (a conventional requirement for all HCSCs), which is a standard requirement for all HCSCs. In 2012, enhanced HCSCs were introduced to provide higher-quality home healthcare, with a focus on emergency home visits and EOL care ^(12)^. Enhanced HCSCs are required to meet the following additional requirements: they should have three or more full-time doctors, conduct at least 10 physician-led emergency home visits in the past year, and provide home-based EOL care in at least four cases during the past year ^(13)^. If a conventional HCSC meets the enhanced requirements, it can be upgraded to receive a higher fee ^(12)^. In addition, patients who receive physician home visits often use nursing care and home-help services offered by various care facilities ^(14)^. Despite the increasing number of HCSCs, general clinics that do not meet the HCSC requirements also provide home visits ^(13)^.

### Long-term care in Japan

In 2000, Japan also introduced a mandatory long-term care (LTC) insurance system distinct from the national medical insurance system ^(15)^. The Japanese LTC insurance system has been described in detail elsewhere ^(16), (17)^. It mandates insurance premiums for all LTC services. All residents aged 40 years and above pay insurance premiums, whereas those aged 65 years and above (and those aged 40-64 years with age-related diseases) are eligible to receive LTC services, including home-, community-, and facility-based care. There are three types of facility-based care services: LTC welfare facilities (i.e., living facilities for patients in a stable condition), LTC health facilities (i.e., intermediate care facilities that aim to discharge individuals needing care and rehabilitation at home), and LTC medical facilities (i.e., medical-based facilities for individuals needing substantial care and long-term treatment) ^(18)^.

### Data source

We sourced our dataset from the MHLW website ^(19)^, where various survey data, including Vital Statistics ^(20)^, Survey of Medical Institutions ^(21)^, and Survey of Institutions and Establishments for Long-term Care ^(22)^, were reaggregated for each municipality. This dataset includes the older population, medical and LTC facility resources, and proportion of home deaths at the municipal level of 1,741 areas. Furthermore, we used original data from the Survey of Medical Institutions ^(21)^ to obtain information on the number of hospital beds. Also, we obtained the population density of the municipalities from the 2020 population census ^(23)^ and the cumulative number of COVID-19 cases from available information on COVID-19 infections ^(2)^.

### Variables

The proportion of home deaths was the number of home deaths out of the total number of deaths annually. We defined the absolute change in the proportion of home deaths by municipality from 2019 to 2021 as the dependent variable. For independent variables, we defined each municipality’s 2019 home death percentage, medical resources (hospital beds, general clinics, conventional HCSCs, enhanced HCSCs, and home-visiting nurses), LTC facility resources (beds in each of the LTC welfare facilities, LTC health facilities, and LTC medical facilities), population density, and cumulative number of COVID-19 cases through December 2021. We used cumulative data on COVID-19 cases at the prefecture level due to the unavailability of municipal-level data. The number of hospital beds was calculated by subtracting the number of psychiatric beds from the total number of beds in all hospitals. We used the 2019 data for all variables except population density, which was obtained from 2020 due to the unavailability of 2019 data. To account for population variations, we adjusted the medical and LTC facility resource variables by dividing them by the number of residents aged 65 years and above in each municipality (shown as “per 10,000 of the municipality-level population” in the tables). The age of 65 years was chosen because (i) more than 90% of deaths occurred in people aged 65 years and above ^(24)^ and (ii) the vast majority (over 95%) of regular home visits were conducted for this age group ^(25)^. The cumulative number of COVID-19 cases was divided by the total population of each prefecture.

### Statistical analysis

First, we examined the trends in the total number of deaths and the proportion of home deaths from 2015 to 2021 across Japan to identify the trend before and after the pandemic. Then, we demonstrated regional variations by depicting the absolute change in the proportion of home deaths from 2019 to 2021, which was the period after the pandemic began. Subsequently, we obtained summary statistics for each variable in 1,741 municipalities. Finally, we conducted a multivariable regression analysis to identify the factors associated with the increase in the proportion of home deaths from 2019 to 2021, including home death percentage in 2019, medical resources (hospital beds, general clinics, conventional HCSCs, enhanced HCSCs, and home-visiting nurses), LTC facility resources (beds in welfare facilities, LTC health facilities, and LTC medical facilities), population density, and cumulative number of COVID-19 cases. The dependent variable was the absolute change in the proportion of home deaths from 2019 to 2021. In this analysis, we included 1,696 municipalities and excluded 45 municipalities where no home deaths occurred in 2019 or 2021. All variables were standardized (mean = 0, standard deviation = 1) before the analyses, and standardized coefficients were interpreted.

Sensitivity analyses were conducted to assess the robustness of the main analyses. First, we conducted a regression analysis by dividing the resources of medical and LTC facilities based on the population aged 75 years and above instead of 65 years as in the main analysis. This decision was made as the primary users of home healthcare and LTC facilities are aged 75 years and above ^(25)^. Second, we conducted a logistic regression analysis, dividing the municipalities into two groups (larger and smaller change groups) based on the absolute change in the proportion of home deaths, using the median value as the cutoff.

As a *post hoc* analysis, we evaluated the correlation between population density and conventional HCSCs as well as between population density and enhanced HCSCs using Spearman’s correlation coefficients.

All the analyses were conducted using STATA version 15 (Stata Corp., Texas, USA). Statistical significance was set at *p* < 0.05.

### Ethical considerations

No ethical approval was obtained as we used only publicly available data.

## Results

### Trends in home deaths

**Figure 1.**
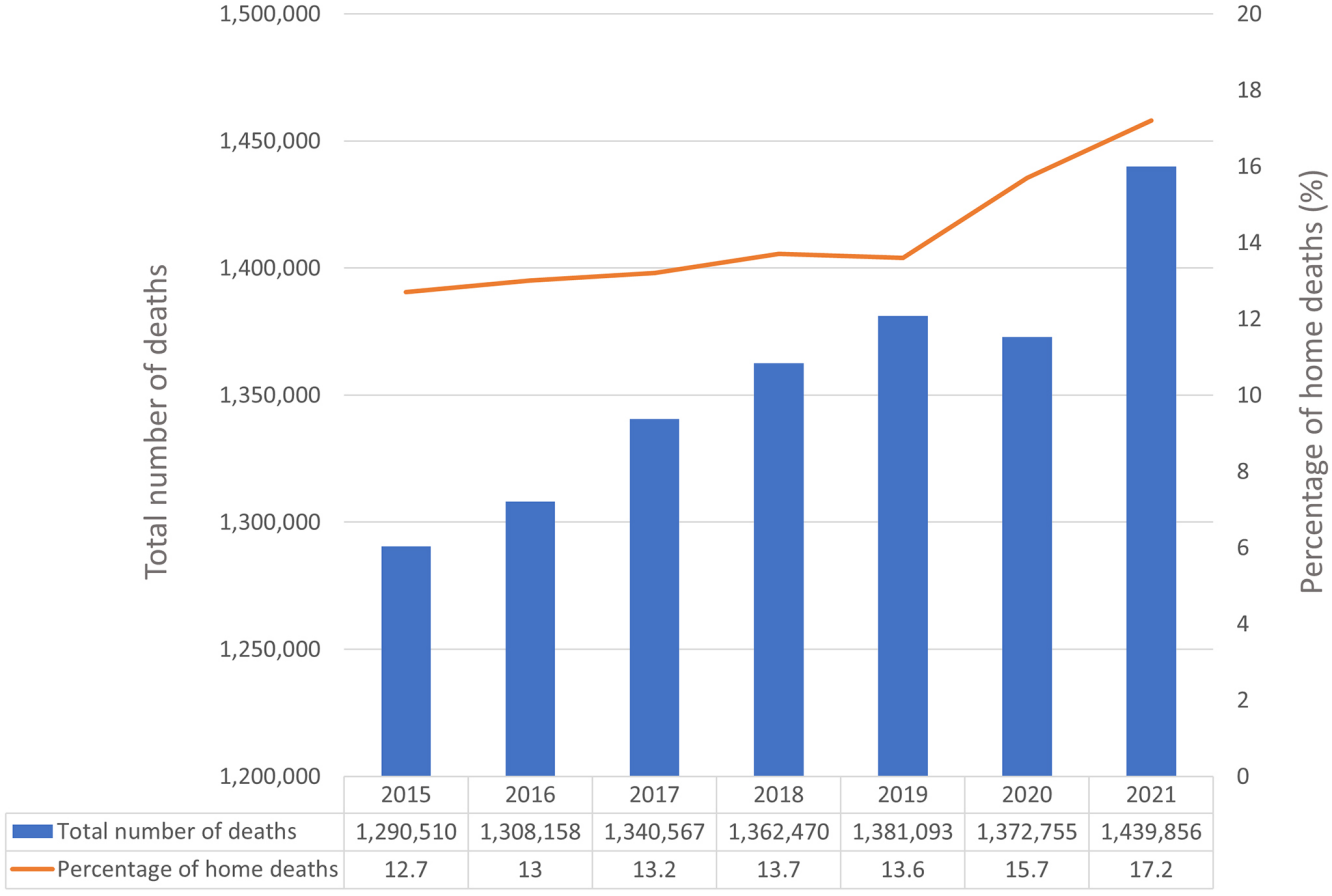
Trends in total deaths and the proportion of home deaths in Japan from 2015 to 2021.

Figure 1 presents the trends in total deaths and proportion of home deaths from 2015 to 2021 in Japan. Between 2015 and 2019, an annual increase of approximately 200,000 deaths was observed, which decreased in 2020 but increased in 2021. The proportions of home deaths in 2015, 2019, and 2021 were 12.7%, 13.6%, and 17.2%, respectively, indicating an increasing trend from 2019 to 2021.

### Regional differences in the increase in home deaths

**Figure 2.**
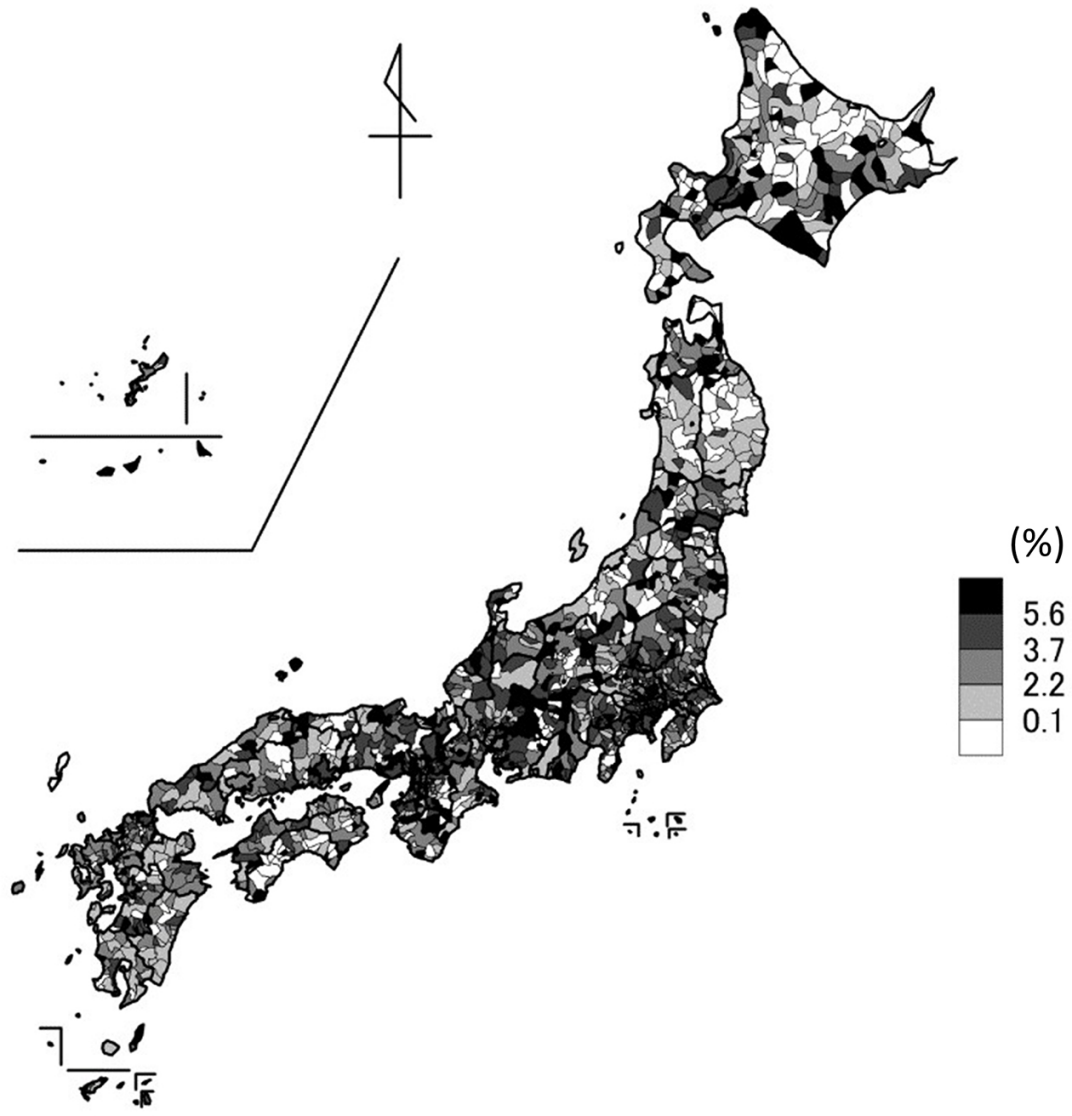
Increase in the proportion of home deaths from 2019 to 2021 at the municipal level.

Figure 2 presents changes in the proportion of home deaths between 2019 and 2021. Notably, the proportion of home deaths was more likely to increase in metropolitan regions, such as Tokyo and Osaka. The distributions of the proportion of home deaths in 2019 and 2021 are presented in Supplementary Appendices 1 and 2, respectively, both of which indicate that home deaths were more prevalent in urban areas.

### Factors associated with the increase in home deaths

Table 1 presents the summary statistics for each variable across the 1,741 municipalities. The distribution of the change in the proportion of home deaths in 1,696 municipalities, excluding those with no home deaths in 2019 or 2021, is presented in Supplementary Appendix 3, indicating a nearly normal distribution. The median and mean absolute changes in the proportion of home deaths were both 2.9%.

**Table 1.** Characteristics of the 1741 Municipalities in Japan.

	Mean (SD)	Median (IQR)
Medical resources*		
Number of hospital beds	2576.1 (2480.7)	2386.6 (0-3736.5)
Number of general clinics	213.7 (213.9)	193.3 (144.9-240.0)
Number of conventional HCSCs	28.8 (45.5)	20.6 (0-40.6)
Number of enhanced HCSCs	6.7 (14.0)	0 (0-9.3)
Number of home-visiting nurses	111.5 (109.4)	100.0 (0-168.1)
LTC resources*		
Number of beds in LTC welfare facilities	220.8 (179.1)	179.0 (130.0-261.3)
Number of beds in LTC health facilities	117.4 (133.2)	101.8 (0-157.9)
Number of beds in LTC medical facilities	10.8 (46.7)	0 (0-2.5)
Population density (population per km^2^)	1069.2 (2607.4)	189.6 (52.3-766.3)
Cumulative number of COVID-19 cases †	10.4 (6.9)	8.3 (5.3-14.2)
Percentage of home deaths in 2019 (%)	11.4 (4.9)	11.1 (8.3-14.1)
Change in the percentage of home deaths between 2019 and 2021 (%)	2.9 (5.2)	2.9 (0.7-5.0)

*All medical and LTC resources are shown per 10,000 people aged 65 years and above. †The cumulative number of COVID-19 cases is shown per 1,000 total population in the prefecture level. Abbreviations: SD, standard deviation; IQR, interquartile range; HCSCs, home care support clinics/hospitals; LTC, long-term care

The results of the multivariable linear regression analysis are shown in Table 2, which indicated that the numbers of conventional HCSCs (coefficient [95% confidence intervals (CIs)] was 0.19 [0.01-0.37]), enhanced HCSCs (0.53 [0.34-0.71]), and home-visiting nurses (0.26 [0.06-0.46]), the population density (0.44 [0.21-0.67]), and the cumulative number of COVID-19 cases (0.49 [0.27-0.70]) were positively associated with the increase in the proportion of home deaths from 2019 to 2021, whereas the number of beds in LTC welfare facilities (−0.55 [−0.74-−0.37]) and proportion of home deaths in 2019 (−1.24 [−1.44-−1.05]) were negatively associated.

**Table 2.** Multivariable Linear Regression Analysis for the Changes in the Proportion of Home Deaths.

	Coefficient	95% CI	*p*-value
Medical resources*			
Number of hospital beds	−0.19	−0.40-0.01	0.066
Number of general clinics	0.15	−0.05-0.34	0.137
Number of conventional HCSCs	0.19	0.01-0.37	0.041
Number of enhanced HCSCs	0.53	0.34-0.71	<0.001
Number of home-visiting nurses	0.26	0.06-0.46	0.011
LTC resources*			
Number of beds in LTC welfare facilities	−0.55	−0.74-−0.37	<0.001
Number of beds in LTC health facilities	0.02	−0.17-0.20	0.872
Number of beds in LTC medical facilities	−0.05	−0.24-0.14	0.609
Population density (population per km^2^)	0.44	0.21-0.67	<0.001
Cumulative number of COVID-19 cases†	0.49	0.27-0.70	<0.001
Percentage of home deaths in 2019 (%)	−1.24	−1.44-−1.05	<0.001

*All medical and LTC resources were divided by 10,000 people aged 65 years and above. †The cumulative number of COVID-19 cases was divided by 1,000 total population in each prefecture. All variables were standardized before analysis, and standardized coefficients were interpreted. Municipalities with no home deaths in 2019 or 2021 were excluded (n = 45). Abbreviations: SD, standard deviation; IQR, interquartile range; HCSCs, home care support clinics/hospitals; LTC, long-term care

The first sensitivity analysis, in which the medical and LTC facility resource variables were divided by the population of residents aged 75 years and above, yielded results consistent with the main analysis (Supplementary Appendix 4). In the second sensitivity analysis, logistic regression analysis using a cutoff of 2.9% yielded similar results, except that there was no statistically significant association for conventional HCSCs and home-visiting nurses but there was a positive association for general clinics (Supplementary Appendices 5 and 6).

### Correlation between the population density and HCSCs

**Figure 3.**
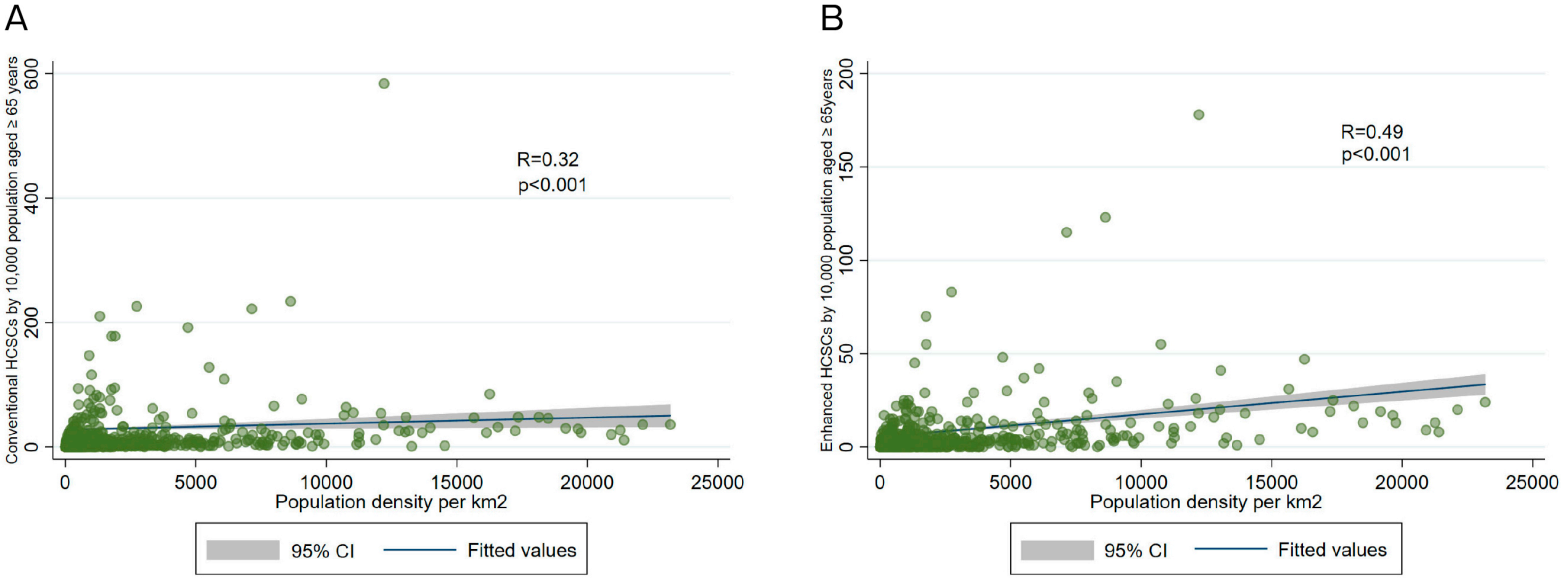
A. Correlation between population density and conventional HCSCs. B. Correlation between population density and enhanced HCSCs.

Figure 3A and 3B present the correlation between population density and conventional and enhanced HCSCs, respectively. Spearman’s correlation coefficients were 0.32 (*p* < 0.001) and 0.49 (*p* < 0.001), respectively, indicating a moderate association between population density and enhanced HCSCs.

## Discussion

To the best of our knowledge, this is the first national-level study to examine trends in home deaths and to identify regional factors associated with increased home deaths during the COVID-19 pandemic in Japan. Our findings show a notable increase in home deaths during this period. Furthermore, we observed positive associations between the increase in home deaths and the number of conventional HCSCs, enhanced HCSCs, home-visiting nurses, population density, and cumulative number of COVID-19 cases. Conversely, we found negative associations between the number of beds in LTC welfare facilities and the proportion of home deaths in 2019.

Our finding of an increased proportion of home deaths during the COVID-19 pandemic is consistent with that of a previous study based on a questionnaire survey conducted in home-visit facilities ^(6)^. While the current data do not allow analysis of the causes of these deaths, a significant increase in the proportion of home deaths directly caused by COVID-19 is unlikely, as COVID-19-related deaths account for only approximately 1% of all deaths in 2021 ^(26)^. By contrast, as reported by previous studies, the shortage of hospital beds and changes in the preferred location for EOL care due to visitation restrictions in inpatient facilities likely played a substantial role ^(6), (7), (8)^.

Regarding regional differences, a notable increase in the proportion of home deaths was observed, particularly in urban areas. Multivariable regression analysis showed an association between this increase and population density. This may be attributed to a higher demand for home-based EOL care in urban areas due to the greater impact of COVID-19, along with supply factors, such as the presence of more medical resources in urban areas capable of providing home-based EOL care. A previous study demonstrated a higher number of in-home service users in urban areas, with community- and facility-based services being more prevalent in rural areas ^(27)^. This regional difference was considered to be associated with regional disparities in the provision of in-home and facility-based services. In densely populated urban areas, the high prices of land pose a challenge to the construction of new facilities; in-home services are more prevalent, with patients benefiting from efficient home visits. Contrarily, rural areas provide favorable conditions for the construction of new facilities while being disadvantageous for home visits ^(27)^.

Our finding that home healthcare resources, such as HCSCs and home visiting nurses, were associated with an increase in the proportion of home deaths is consistent with those of previous studies conducted before the COVID-19 pandemic ^(28), (29)^. However, our study distinguished between conventional and enhanced HCSCs, showing a stronger association between enhanced HCSCs. Furthermore, in logistic regression analysis, only enhanced HCSCs were associated with an increase in the proportion of home deaths. One possible explanation for this finding is that conventional HCSCs often consist of sole practitioners who provide home healthcare as an adjunct to outpatient services ^(30)^, making it difficult to expand the provision of home healthcare. Conversely, enhanced HCSCs, which require a minimum of three full-time physicians, may be better equipped to increase the number of patients receiving EOL care during a pandemic.

Our finding that the number of LTC welfare facilities is negatively associated with deaths at home is consistent with those of previous studies ^(28), (29)^, and it is reasonable to assume that having a sufficient number of LTC facilities may reduce the likelihood of home deaths. Notably, among the three LTC facilities, only welfare facilities were negatively associated with home deaths. This can be explained by the fact that LTC welfare facilities admit patients with higher care needs and have the highest number of beds among the three types of facilities ^(31)^.

This pandemic has highlighted the importance of establishing a healthcare system that can effectively respond to unusual circumstances. Furthermore, it underscores the significance of having sufficient home healthcare resources to accommodate patients who cannot receive care in hospitals or who have changed their preferences for EOL care settings. However, the study results indicated disparities in home healthcare resources, particularly in enhanced HCSCs, depending on the population density. Areas with low population densities pose challenges for home visits due to the scattered nature of residences, making it time-consuming to move between patients’ homes and limiting the number of patients served ^(30)^. Consequently, areas with low population densities are likely to have fewer enhanced HCSCs, which play a pivotal role in EOL care. To flexibly reinforce the role of home healthcare in unusual situations, policy considerations, such as the implementation of additional fees for enhanced HCSCs based on population density, may be necessary.

This study has several limitations. First, the classification of “death at home” includes not only in patients’ own homes but also in group homes for older people with dementia and elderly housing with care services* [Sabisutsuki koreisya muke Jutaku] *^(32)^. We could not distinguish them due to the lack of a detailed place of death classification in the Vital Statistics. Additionally, “death at home” does not necessarily indicate that the patient received home healthcare; it could include suicide, homicide, or unexpected sudden deaths. However, given that deaths caused by external causes account for only approximately 6% of all deaths at home ^(33)^, this bias is unlikely to substantially affect our results. Second, medical and LTC facility resources may have been affected by neighboring municipalities, which we could not account for. In regions with insufficient HCSCs, neighboring municipalities with HCSCs may influence the likelihood of home death. Third, there might be unmeasured confounders such as patients’ and their families’ preferences, accessibility, and socioeconomic factors. Fourth, because our findings were based on observational data, it was difficult to determine the causal relationships. Abundant local healthcare resources may allow more people to receive EOL care at home, whereas healthcare resources supporting home care may exist in areas where many people already receive care at home. However, the fact that the desire to die at home did not greatly vary according to the population size of the municipality, as reported in a prepandemic study ^(34)^, and the nationwide implementation of visitation restrictions during the pandemic ^(5)^, suggests a high preference for EOL care at home throughout the country during the pandemic. Thus, it is reasonable to assume that the higher availability of resources for home healthcare was associated with an increased number of home deaths.

In conclusion, using comprehensive national empirical data, including data from the COVID-19 pandemic period in Japan, we observed an increase in the proportion of home deaths after 2019. Such an increase was particularly notable in areas with high population density, significant cumulative number of COVID-19 cases, and robust resources for home healthcare. Notably, we found a moderate correlation between population density and enhanced HCSC, which was the most influential factor in the increase in home deaths. Given the disadvantages faced by areas with low population density in terms of home healthcare, it may be necessary to formulate policies that consider population density to strengthen the role of home healthcare services nationwide during unusual situations.

## Article Information

### Conflicts of Interest

None

### Sources of Funding

This work was supported by the Ministry of Health, Labor and Welfare; Health, Labor and Welfare Sciences Research Grant, Japan; Research on Region Medical (21IA1010).

### Acknowledgement

We would like to thank Editage (www.editage.jp) for its English language editing.

### Author Contributions

Study concept and design: Yu Sun and Masao Iwagami. Statistical analysis and interpretation of data: Yu Sun, Masao Iwagami. Preparation of the manuscript: All authors contributed to drafting and critical revision of the manuscript. All authors have approved the final manuscript.

## Supplement

Supplementary Appendices Supplementary Appendix 1. Proportion of deaths at home in 2019 at the municipal level
Supplementary Appendix 2. Proportion of deaths at home in 2021 at the municipal level
Supplementary Appendix 3. Distribution of the change in the proportion of deaths at home in 1,696 municipalities
Supplementary Appendix 4. Multivariable linear regression analysis for changes in the proportion of home deaths with medical and LTC resources divided by 75 years and above
Supplementary Appendix 5. Characteristics of the absolute change in home deaths rates >2.9% and ≤2.9%
Supplementary Appendix 6. Multivariable logistic regression analysis for changes in the proportion of home deaths with cutoff set at 2.9%

## References

[1] Zhu N, Zhang D, Wang W, et al. A novel coronavirus from patients with pneumonia in China, 2019. N Engl J Med. 2020;382(8):727-33. 31978945 10.1056/NEJMoa2001017 PMC7092803

[2] Japanese Ministry of Health, Labour and Welfare. Visualizing the data: information on COVID-19 infections. [Internet]. [cited 2023 Dec 8]. Available from: https://covid19.mhlw.go.jp/en/.

[3] Kurahara Y, Kobayashi T, Shintani S, et al. Clinical characteristics of COVID-19 in Osaka, Japan: comparison of the first-third waves with the fourth wave. Respir Investig. 2021;59(6):810-8. 10.1016/j.resinv.2021.08.005 PMC8429365 34565716

[4] Japanese Ministry of Health, Labour and Welfare. Results of a survey on the treatment status of COVID-19 patients, number of hospital beds, etc. [Internet]. [cited 2023 Dec 8]. Available from: https://www.mhlw.go.jp/stf/seisakunitsuite/newpage_00023.html. Japanese.

[5] Results of the second questionnaire survey on the impact of COVID-19 in palliative care units. Hospice Palliative Care Japan [Internet]. [cited 2023 Oct 5]. Available from: https://www.hpcj.org/info/covid19/covid19_pcuchosa202111.pdf. Japanese.

[6] Hamano J, Tachikawa H, Takahashi S, et al. Changes in home visit utilization during the COVID-19 pandemic: a multicenter cross-sectional web-based survey. BMC Res Notes. 2022;15(1):238. 35799212 10.1186/s13104-022-06128-7 PMC9261221

[7] Imanaga T. The association between the proportion of deaths at home in cancer patients and the number of COVID-19 infected persons. J Jpn Assoc Home Care Med. 2023;4(2):1-6. Japanese.

[8] O’Donnell SB, Bone AE, Finucane AM, et al. Changes in mortality patterns and place of death during the COVID-19 pandemic: A descriptive analysis of mortality data across four nations. Palliat Med. 2021;35(10):1975-84. 34425717 10.1177/02692163211040981 PMC8641034

[9] Bone AE, Finucane AM, Leniz J, et al. Changing patterns of mortality during the COVID-19 pandemic: population-based modelling to understand palliative care implications. Palliat Med. 2020;34(9):1193-201. 32706299 10.1177/0269216320944810 PMC7385436

[10] Ikegami N, Yoo BK, Hashimoto H, et al. Japanese universal health coverage: evolution, achievements, and challenges. Lancet. 2011;378(9796):1106-15. 21885107 10.1016/S0140-6736(11)60828-3

[11] Japanese Ministry of Health, Labour and Welfare. An outline of the Japanese medical system. [Internet]. [cited 2023 Dec 8]. Available from: https://www.mhlw.go.jp/english/policy/health-medical/health-insurance/index.html.

[12] Ohta H. Current conditions and issues for home care support clinics. Japan Med Assoc J. 2015;58(1-2):6-9. PMC4597930 26557454

[13] Japanese Ministry of Health, Labour and Welfare. General meeting materials of central social insurance medical council 2019. [Internet]. [cited 2023 Dec 8]. Available from: https://www.mhlw.go.jp/content/12404000/000563523.pdf. Japanese.

[14] Fukui S, Yamamoto-Mitani N, Fujita J. Five types of home-visit nursing agencies in Japan based on characteristics of service delivery: cluster analysis of three nationwide surveys. BMC Health Serv Res. 2014;14:644. 25527199 10.1186/s12913-014-0644-8 PMC4301826

[15] Tamiya N, Noguchi H, Nishi A, et al. Population ageing and wellbeing: lessons from Japan's long-term care insurance policy. Lancet. 2011;378(9797):1183-92. 21885099 10.1016/S0140-6736(11)61176-8

[16] Tsutsui T, Muramatsu N. Care-needs certification in the long-term care insurance system of Japan. J Am Geriatr Soc. 2005;53(3):522-7. 15743300 10.1111/j.1532-5415.2005.53175.x

[17] Houde SC, Gautam R, Kai I. Long-term care insurance in Japan: implications for U.S. long-term care policy. J Gerontol Nurs. 2007;33(1):7-13. 10.3928/00989134-20070101-04 17305264

[18] Iwagami M, Tamiya N. The long-term care insurance system in Japan: past, present, and future. JMA J. 2019;2(1):67-9. 33681515 10.31662/jmaj.2018-0015 PMC7930803

[19] Japanese Ministry of Health, Labour and Welfare. Regional data collection for home healthcare. [Internet]. [cited 2023 Dec 8]. Available from: https://view.officeapps.live.com/op/view.aspx?src=https%3A%2F%2Fwww.mhlw.go.jp%2Fcontent%2F10800000%2F001094335.xlsx&wdOrigin=BROWSELINK. Japanese.

[20] Japanese Ministry of Health, Labour and Welfare. Outline of vital statistics in Japan. [Internet]. [cited 2023 Dec 8]. Available from: https://www.mhlw.go.jp/english/database/db-hw/outline/index.html.

[21] Japanese Ministry of Health, Labour and Welfare. Survey of medical institutions. [Internet]. [cited 2023 Dec 8]. Available from: https://www.mhlw.go.jp/english/database/db-hss/smi.html.

[22] Japanese Ministry of Health, Labour and Welfare. Survey of institutions and establishments for long-term care. [Internet]. [cited 2023 Dec 8]. Available from: https://www.mhlw.go.jp/english/database/db-hss/siel-index.html.

[23] Statistics Bereau of Japan. Population census. [Internet]. [cited 2023 Dec 8]. Available from: https://www.stat.go.jp/english/data/kokusei/index.html.

[24] Statistics of Japan. Number of deaths and death rates by age. [Internet]. [cited 2023 Dec 8]. Available from: https://www.e-stat.go.jp/dbview?sid=0003411690. Japanese.

[25] Japanese Ministry of Health, Labour and Welfare. Reference materials for the 1st National Conference on home medical care. 2016. [Internet]. [cited 2023 Dec 8]. Available from: https://www.mhlw.go.jp/file/05-Shingikai-10801000-Iseikyoku-Soumuka/0000129546.pdf. Japanese.

[26] Tanaka, H, Togawa K, Katanoda K. Impact of the COVID-19 pandemic on mortality trends in Japan: a reversal in 2021? A descriptive analysis of national mortality data, 1995-2021. BMJ Open. 2023;13(8):e071785. 10.1136/bmjopen-2023-071785 PMC10476106 37652585

[27] Okada R, Goto E, Shin J, et al. Regional differences and related factors in the use of long-term care insurance services by municipality. J Jpn Soc Healthc Admin. 2023;60:44-52. Japanese.

[28] Morioka N, Tomio J, Seto T, et al. Association between local-level resources for home care and home deaths: A nationwide spatial analysis in Japan. PLOS ONE. 2018;13(8):e0201649. 30142197 10.1371/journal.pone.0201649 PMC6108466

[29] Ikeda T, Tsuboya T. Place of death and density of homecare resources: A nationwide study in Japan. Ann Geriatr Med Res. 2021;25(1):25-32. 33794586 10.4235/agmr.21.0003 PMC8024167

[30] Japan Medical Association Research Institute. 2nd survey of home health care function of clinics [Internet]. 2017. [cited 2023 Dec 8]. Available from: http://www.jmari.med.or.jp/download/WP392.pdf. Japanese.

[31] Igarashi A, Eltaybani S, Takaoka M, et al. Quality assurance in long-term care and development of quality indicators in Japan. Gerontol Geriatr Med. 2020;6:2333721420975320. 35047653 10.1177/2333721420975320 PMC8762483

[32] Sugimoto K, Ogata Y, Kashiwagi M. Factors promoting resident deaths at aged care facilities in Japan: a review. Health Soc Care Community. 2018;26(2):e207-24. 27696541 10.1111/hsc.12383

[33] Taniguchi Y, Watanabe T, Midorikawa H, et al. Percentage of deaths from external causes among deaths at home by municipality. J Health Welf Stat. 2020;67(3):13-6. Japanese.

[34] Koshida M, Fujimura K, Doi H. Factors related to decision-making on the place of end-of-Life care by population size. Jpn J Health Hum Ecol. 2022;88(5):19fi203. Japanese.

