# Supplementary material for: Change in the Proportion of Death at Home during the COVID-19 Pandemic and Its Associated Factors in the Municipality Level: A Nationwide Study in Japan
Source: JMA J. 2024 Mar 18;7(2):213–21. doi: 10.31662/jmaj.2023-0165 (PMC11074502; doi:10.31662/jmaj.2023-0165)
Supplement: Supplementary Appendices — Supplementary Appendix 1. Proportion of deaths at home in 2019 at the municipal level Supplementary Appendix 2. Proportion of deaths at home in 2021 at the municipal level Supplementary Appendix 3. Distribution of the change in the proportion of deaths at home in 1,696 municipalities Supplementary Appendix 4. Multivariable linear regression analysis for changes in the proportion of home deaths with medical and LTC resources divided by 75 years and above Supplementary Appendix 5. Characteristics of the absolute change in home deaths rates >2.9% and ≤2.9% Supplementary Appendix 6. Multivariable logistic regression analysis for changes in the proportion of home deaths with cutoff set at 2.9% [file 2433-3298-7-2-0213-s001.pdf]

Supplementary Appendix 1. Proportion of deaths at home in 2019 at the municipal level

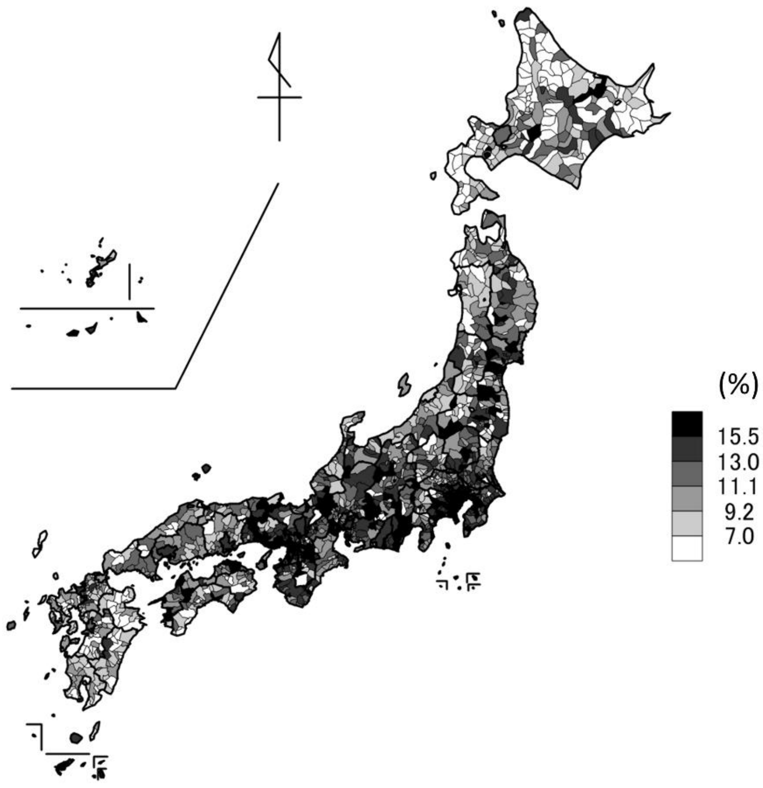

Supplementary Appendix 2. Proportion of deaths at home in 2021 at the municipal level

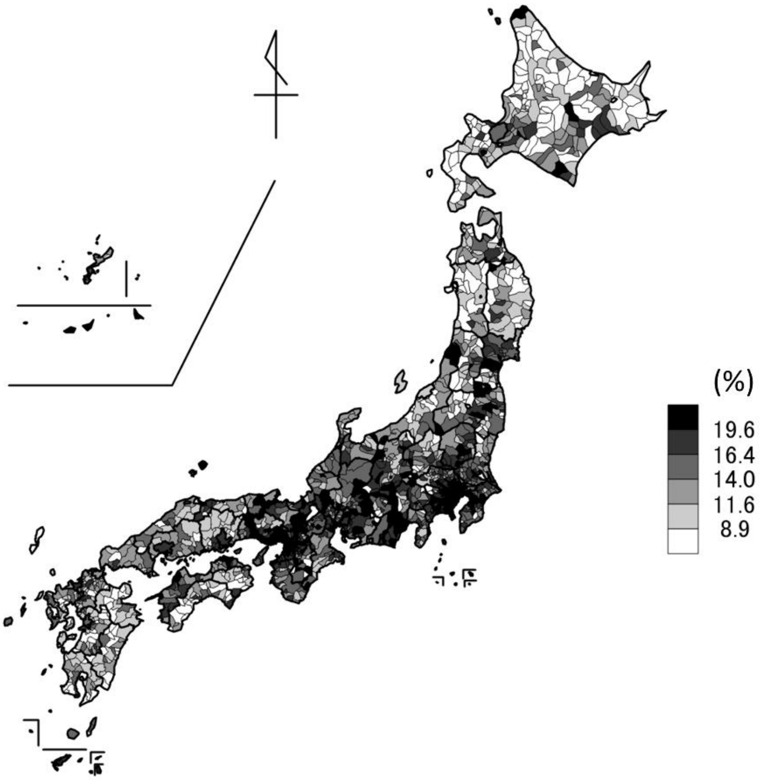

Supplementary Appendix 3. Distribution of the change in the proportion of deaths at home in 1,696 municipalities

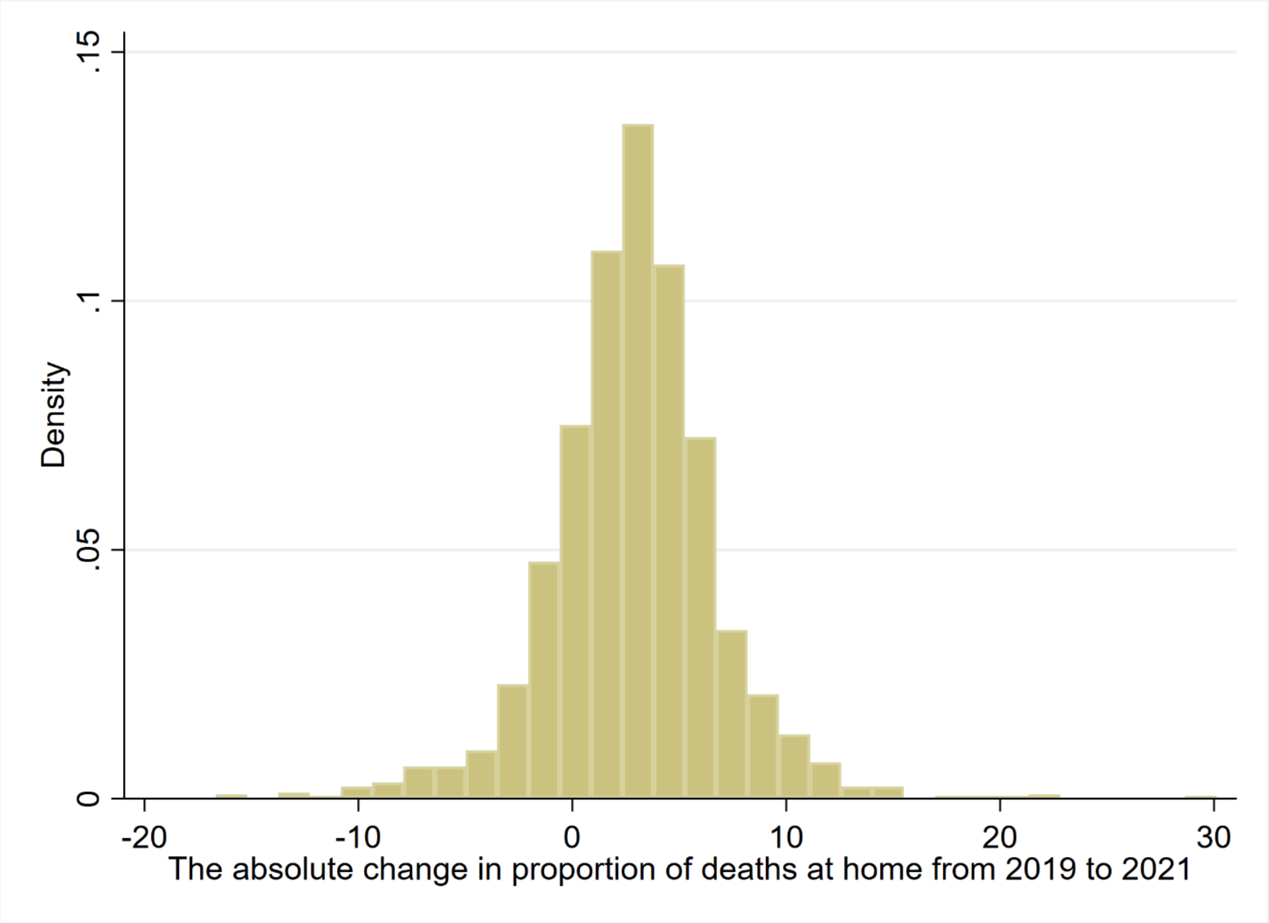

Supplementary Appendix 4. Multivariable linear regression analysis for changes in the proportion of home deaths with medical and LTC resources divided by 75 years and above

	Coefficient	95%CI	p-value
Medical resources*			
The number of hospital beds	−1.73	−0.38 to 0.04	0.103
The number of general clinics	0.17	−0.03 to 0.37	0.087
The number of conventional HCSCs	0.23	0.05 to 0.41	0.012
The number of enhanced HCSCs	0.53	0.34 to 0.72	<0.001
The number of home visiting nurses	0.27	0.07 to 0.47	0.009
LTC resources*			
The number of beds in LTC welfare facilities	−0.50	−0.68 to −0.31	<0.001
The number of beds in LTC health facilities	0.06	−0.12 to 0.24	0.514
The number of beds in LTC medical facilities	−0.06	-0.25 to 0.12	0.499
Population density (population per km^2)	0.43	0.19 to 0.66	<0.001
Cumulative number of COVID-19 cases †	0.48	0.26 to 0.69	<0.001
The percentage of home deaths in 2019 (%)	−1.24	−1.43 to −1.04	<0.001

*All medical and LTC resources were divided among 10,000 people aged 75 years and older.

† The cumulative number of COVID-19 cases was divided by 1,000 total population in each prefecture.

All variables were standardized prior to analysis, and standardized coefficients were interpreted. Municipalities with no home deaths in 2019 or 2021 were excluded from the analysis (n=1,696).

Abbreviations: HCSCs, home care support clinics/hospitals; LTC, long-term care.

Supplementary Appendix 5. Characteristics of the absolute change in home deaths rates >2.9% and ≤2.9%

	Absolute change in deaths rate ≤2.9%	Absolute change in deaths rate >2.9%
	n=847	n=849
	Median (IQR)	Median (IQR)
Medical resources*		
The number of hospital beds	2368.6 (0–3779.6)	2468.0 (959.6–3724.5)
The number of general clinics	181.5 (135.4–230.2)	201.5 (152.8–247.6)
The number of conventional HCSCs	19.3 (0–40.6)	22.6 (0–41.3)
The number of enhanced HCSCs	0 (0–5.9)	0 (0–13.5)
The number of home visiting nurses	88.5 (0–151.7)	119.0 (45.9–184.8)
LTC resources*		
The number of beds in LTC welfare facilities	193.5 (142.9–280.4)	166.6 (125.3–236.6)
The number of beds in LTC health facilities	113.1 (0–169.9)	96.3 (42.2–149.7)
The number of beds in LTC medical facilities	0 (0–4.5)	0 (0–2.7)
Population density (population per km^2)	136.1 (43.2–371.9)	344.4 (81.3–1394.7)
Cumulative number of COVID-19 cases †	7.5 (4.8–11.9)	9.0 (5.8–14.6)
The percentage of home deaths in 2019 (%)	11.0 (8.5–14.0)	11.5 (8.3–14.3)
Change in the percentage of home deaths between 2019 and 2021 (%)	0.8 (−1.0–2.0)	5.0 (3.8–6.6)

*All medical and LTC resources are shown per 10,000 people aged 65 years and older.

†The cumulative number of COVID-19 cases is shown per 1,000 total population in prefecture level.

Abbreviations: IQR, interquartile range; HCSCs, home care support clinics/hospitals; LTC, long-term care

Supplementary Appendix 6. Multivariable logistic regression analysis for changes in the proportion of home deaths with cutoff set at 2.9%

	Odds ratio	95%CI	p-value
Medical resources*			
The number of hospital beds	0.93	0.82–1.04	0.205
The number of general clinics	1.26	1.04–1.54	0.021
The number of conventional HCSCs	1.01	0.91–1.12	0.865
The number of enhanced HCSCs	1.33	1.19–1.50	<0.001
The number of home visiting nurses	1.10	0.98–1.24	0.114
LTC resources*			
The number of beds in LTC welfare facilities	0.82	0.74–0.92	<0.001
The number of beds in LTC health facilities	0.98	0.88–1.08	0.661
The number of beds in LTC medical facilities	0.99	0.89–1.11	0.898
Population density (population per km^2)	1.66	1.34–2.05	<0.001
Cumulative number of COVID-19 cases†	1.26	1.11–1.43	<0.001
The percentage of home deaths in 2019 (%)	0.66	0.59–0.76	<0.001

*All medical and LTC resources were divided by 10,000 people aged 65 years and older.

†The cumulative number of COVID-19 cases was divided by 1,000 total population in each prefecture.

All variables were standardized prior to the analysis. Municipalities with no home deaths in 2019 or 2021 were excluded from the analysis (n=1,696).

Abbreviations: SD, standard deviation; IQR, interquartile range; HCSCs, home care support clinics/hospitals; LTC, long-term care
